# Supplementary material for: Pleiotropic effects between statin intake and inflammation parameters in two distinct population-based studies
Source: Commun Med (Lond). 2025 Sep 11;5:387. doi: 10.1038/s43856-025-01124-x (PMC12426230; doi:10.1038/s43856-025-01124-x)
Supplement: Supplementary file 2 — Supplementary Information [file 43856_2025_1124_MOESM2_ESM.pdf]

Pleiotropic effects between statin intake and inflammation parameters in two distinct population-based studies

Dennis Freuer, Jakob Linseisen, Timo Schmitz, Barbara Thorand, Annette Peters, Agnese Petrera, Margit Heier, Christa Meisinger

Table of contents

Tables

Table 1	Comparison of cohorts between KORA-Fit and KORA-Age1 studies
Table 2	E-values for revealed associations in KORA-Fit and KORA-Age1 studies

Figures

Figure 1	Directed acyclic graph for confounder selection
Figure 2	Estimates obtained from linear regression models in the KORA-Fit study
Figure 3	Estimates obtained from log-linear regression models in the KORA-Fit study
Figure 4	Estimates obtained from linear regression models in the KORA-Age1 study
Figure 5	Estimates obtained from log-linear regression models in the KORA-Age1 study

Supplementary Table 1: Comparison of the cohorts between the two studies KORA-Fit and KORA-Age1

Characteristic	KORA-Age1 (n=1079)	KORA-Fit (n=856)	*P*
Age (years)	76 (70; 81)	63 (58; 67)	0
BMI (kg/m^2)	27.88 (25.37; 30.74)	27.59 (24.36; 31.188)	0.03
Alcohol consumption (g/day)	5.71 (0; 20)	5.71 (0; 22.812)	0.005
Systolic blood pressure (mmHg)	137 (124.5; 150)	123 (113.5; 134)	0
Cholesterol (mg/dl)	209 (182; 236)	212 (183.225; 238)	0.233
Triglycerides (mg/dl)	123.5 (89; 176.75)	107 (77; 150.475)	0
HDL cholesterol (mg/dl)	54 (45; 64)	61.1 (49; 75.33)	0
LDL cholesterol (mg/dl)	125 (104; 150)	127 (101; 151.25)	0.646
Non-HDL cholesterol (mg/dl)	153 (127; 176)	146.02 (120.075; 174.125)	0.001
Sex			0.102
men	537 (0.498)	394 (0.46)	
women	542 (0.502)	462 (0.54)	
Education (years)			0
[08; 10]	269 (0.249)	371 (0.433)	
[11; 13]	117 (0.108)	173 (0.202)	
[14; 17]	693 (0.642)	312 (0.364)	
Statin use			0
yes	296 (0.274)	138 (0.161)	
no	783 (0.726)	717 (0.839)	
Stroke			0
yes	95 (0.088)	23 (0.027)	
no	984 (0.912)	833 (0.973)	
Physical activity			0
regular (> 2h)	304 (0.282)	321 (0.375)	
regular (1h)	260 (0.241)	280 (0.327)	
unregular (1h)	144 (0.134)	108 (0.126)	
little or not	370 (0.343)	147 (0.172)	
Smoking status			0
current	49 (0.045)	119 (0.139)	
previous	448 (0.415)	369 (0.432)	
never	582 (0.539)	366 (0.429)	
Myocardial infarction			0
yes	111 (0.103)	26 (0.03)	
no	968 (0.897)	830 (0.97)	
Cancer			0.16
yes	152 (0.141)	102 (0.119)	
no	927 (0.859)	754 (0.881)	

Continuous variables are reported as median and interquartile range and tested with the Mann-Whitney U test. Categorical variables are presented as absolute and relative frequencies and tested using the χ^2-test.

Abbreviations: BMI, body mass index; HbA1c, Hemoglobin A1c; HDL, high-density lipoprotein; KORA, Cooperative Health Research in the region of Augsburg; LDL, low-density lipoprotein

Supplementary Table 2: E-values for revealed associations in KORA-Fit and KORA-Age1 studies

Outcome	E-value Beta	E-value CI	Strongest Association	Strongest Beta
		**KORA-Fit**		
vegfa	1,71	1,08	phys_actunregular_1h	0,17
trail	2,02	1,48	diabetno	0,11
scf	1,91	1,36	smokernever	0,25
trance	2,08	1,53	rcs(bmi, 4)bmi''	0,48
cd40	1,73	1,11	diabetno	0,08
fgf19	1,76	1,17	diabetno	0,27
nt3	2,15	1,60	educ[8;10]	0,22
slamf1	1,73	1,12	phys_actunregular_1h	0,07
ada	1,85	1,29	educ[8;10]	0,02
		**KORA-Age1**		
cd244	1,82	1,45	smokernever	0,14
upa	1,74	1,36	phys_actunregular_1h	0,07
trail	2,03	1,66	statin_useno	0,13
il2	1,63	1,22	phys_actlittle_or_not	0,05
scf	1,92	1,55	smokernever	0,12
il18	1,63	1,23	phys_actlittle_or_not	0,23
mmp1	1,67	1,28	phys_actlittle_or_not	0,43
pdl1	1,72	1,34	rcs(bmi, 4)bmi'	0,44
trance	1,78	1,41	rcs(alkkon, 5)alkkon''	0,28
mmp10	1,84	1,47	phys_actlittle_or_not	0,22
gdnf	1,71	1,33	phys_actlittle_or_not	0,12
cxcl11	1,55	1,10	phys_actlittle_or_not	0,04
il20ra	1,26	1,06	phys_actunregular_1h	0,23
mcp4	1,54	1,05	phys_actlittle_or_not	0,02
il24	1,58	1,14	medic_antihyperno	0,10
il13	1,27	1,05	educ[14;17]	0,68
cxcl6	1,61	1,20	phys_actlittle_or_not	0,04

E-values quantify the minimum strength (on the risk ratio scale) of an observed confounder to explain away the specific association.

Abbreviations: CI, confidence interval

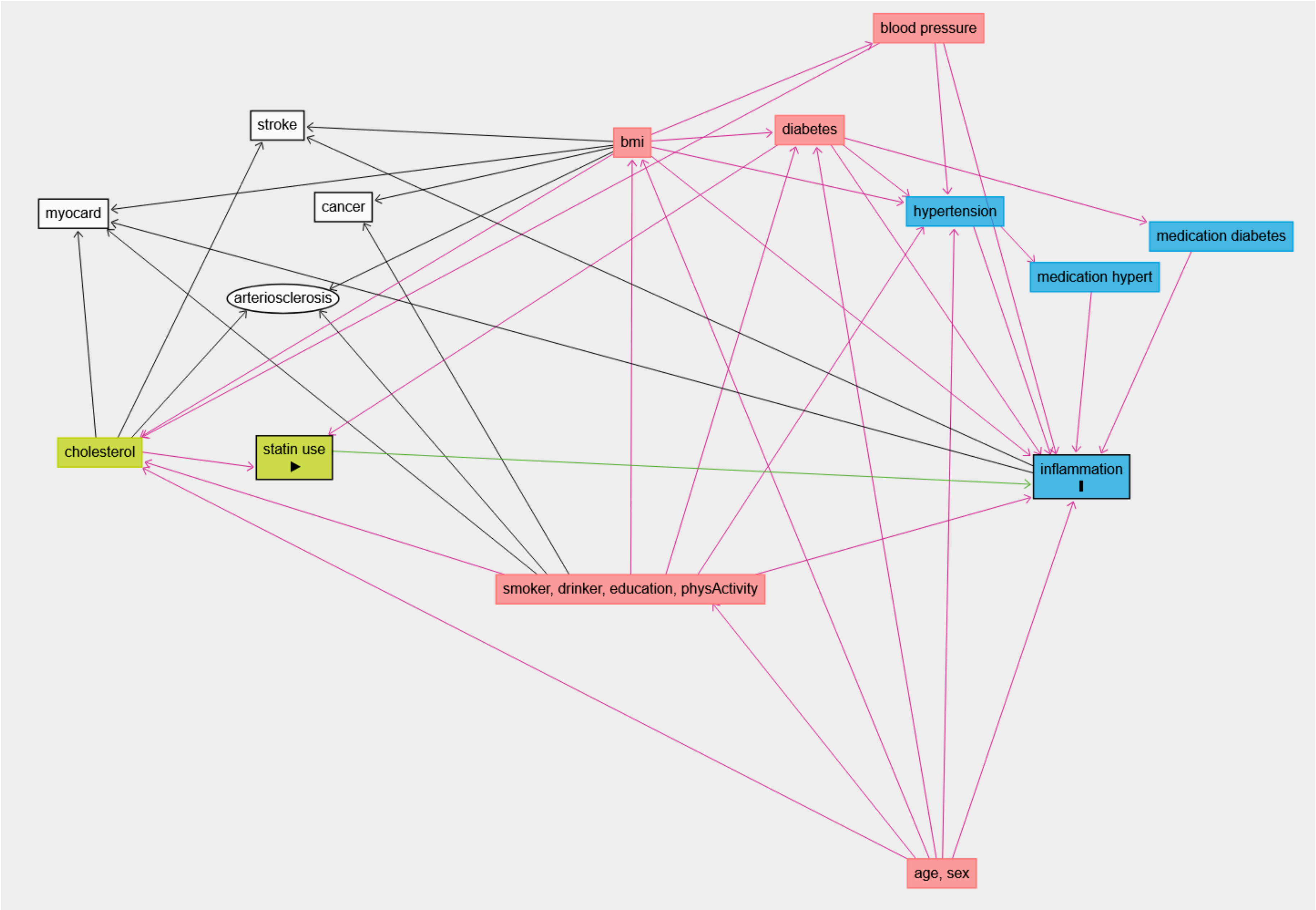

Supplementary Figure 1: Directed acyclic graph used for confounder selection.
Confounders and mediators are colored red and blue, respectively. Colliders are uncolored. Cholesterol is an instrument of statin intake.

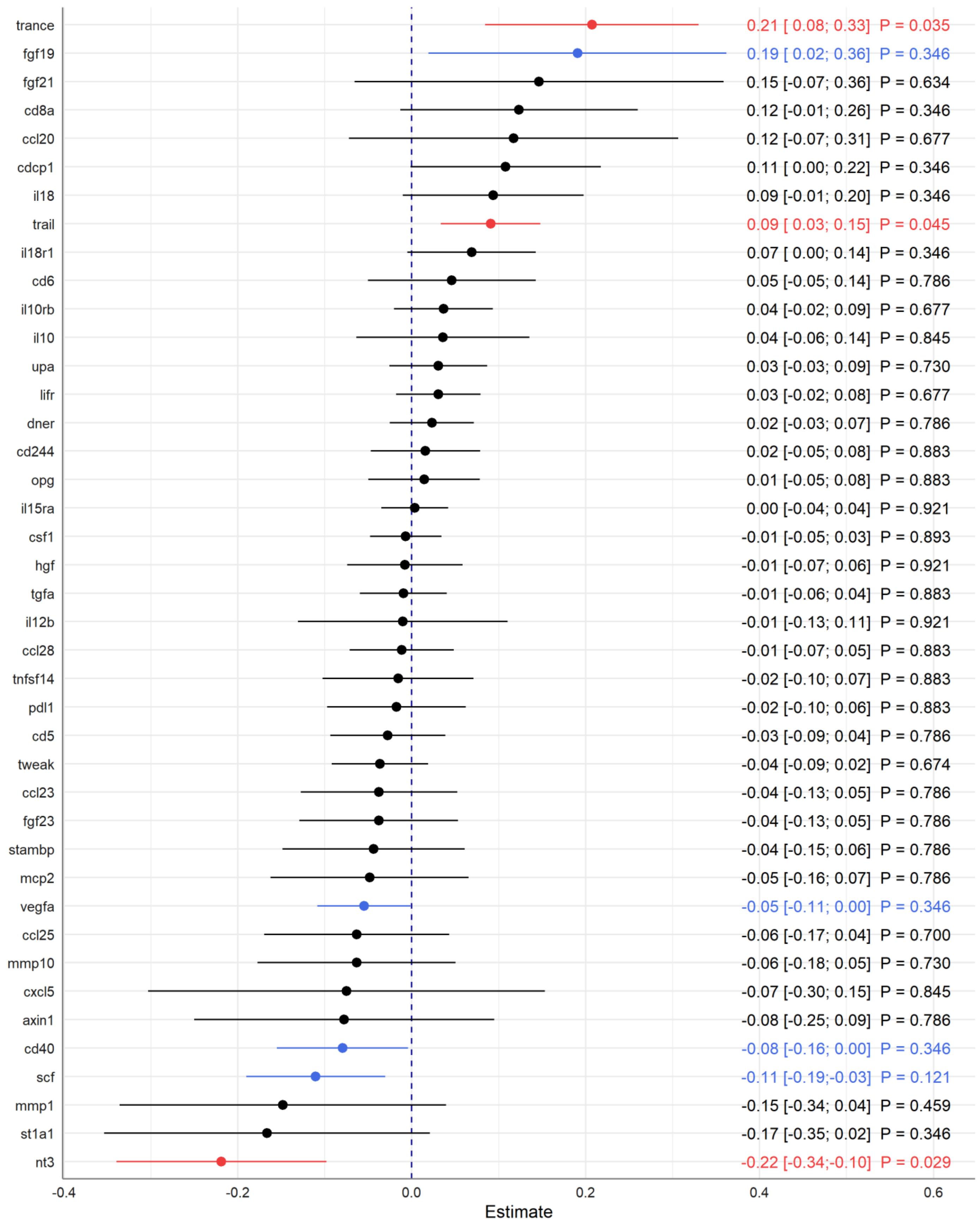

Supplementary Figure 2: Estimates obtained from multivariable linear regression models in the KORA-Fit study. β coefficients and 95% confidence intervals represent the associations between statin intake and inflammation parameters. P-values are FDR-adjusted. Red and blue colors represent notable associations before and after adjustment for multiple testing.

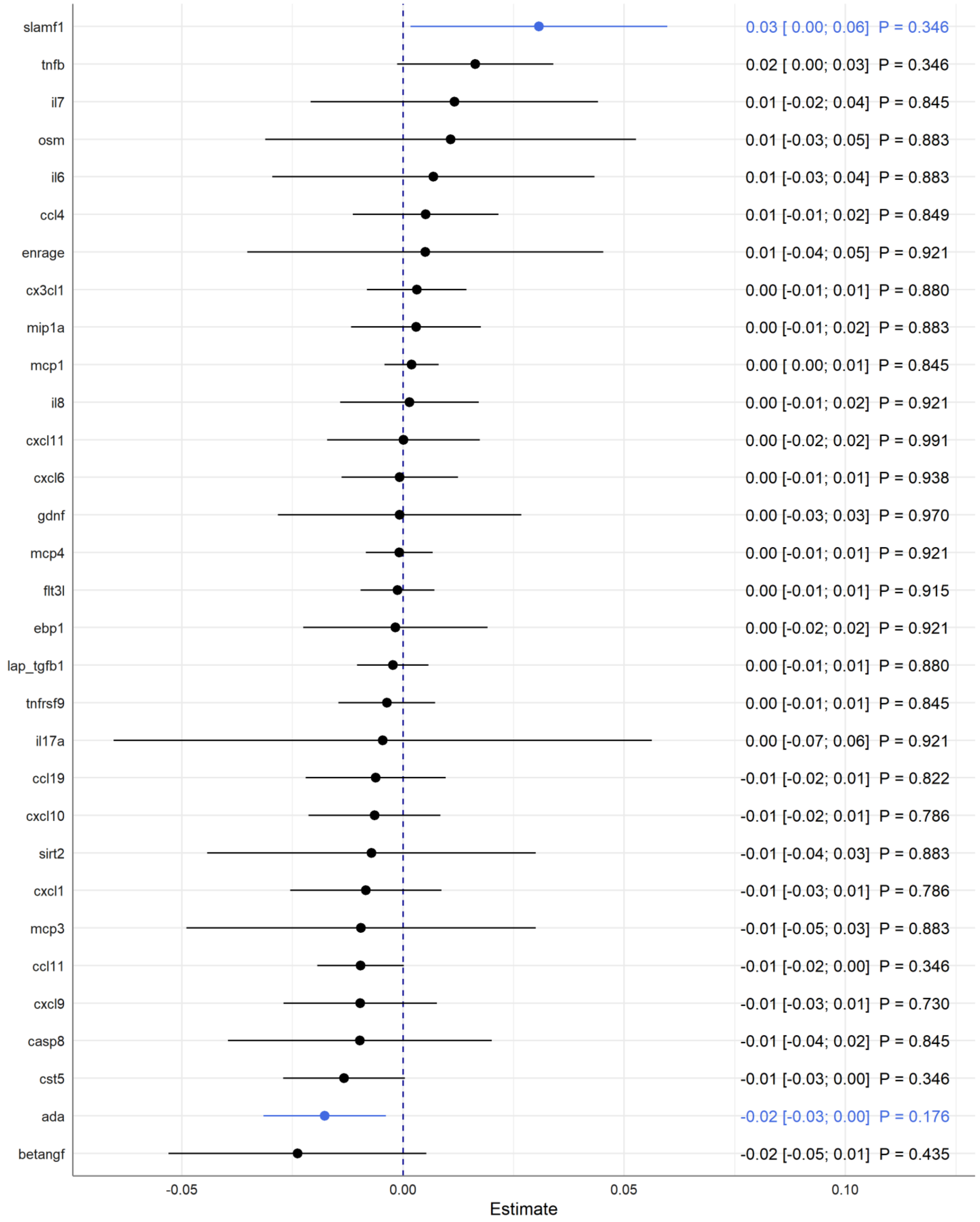

Supplementary Figure 3: Estimates obtained from multivariable log-linear regression models in the KORA-Fit study.

β coefficients and 95% confidence intervals represent the associations between statin intake and inflammation parameters. P-values are FDR-adjusted. Blue colored estimates represent notable associations before adjustment for multiple testing.

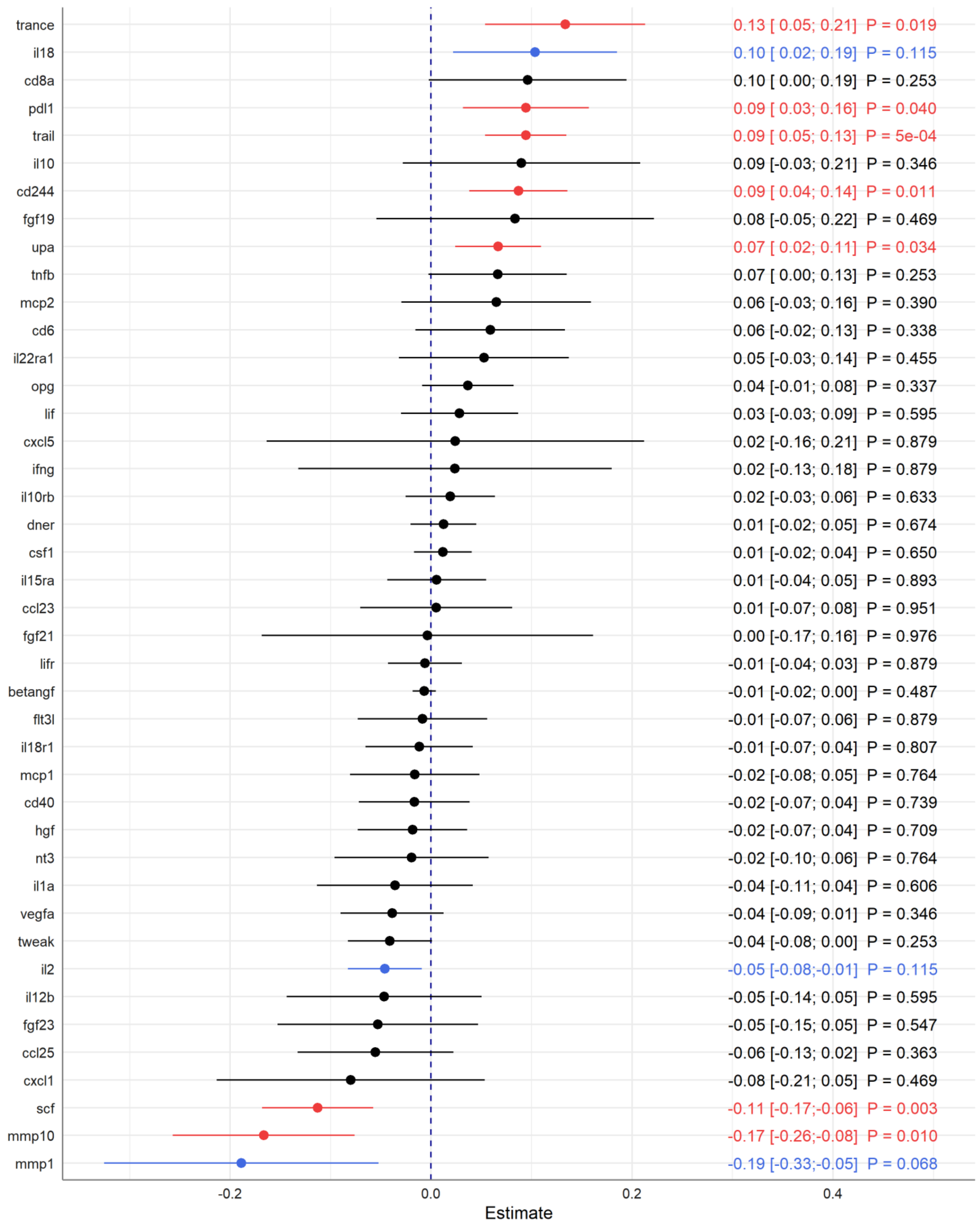

Supplementary Figure 4: Estimates obtained from multivariable linear regression models in the KORA-Age1 study. β coefficients and 95% confidence intervals represent the associations between statin intake and inflammation parameters. P-values are FDR-adjusted. Red and blue colors represent notable associations before and after adjustment for multiple testing.

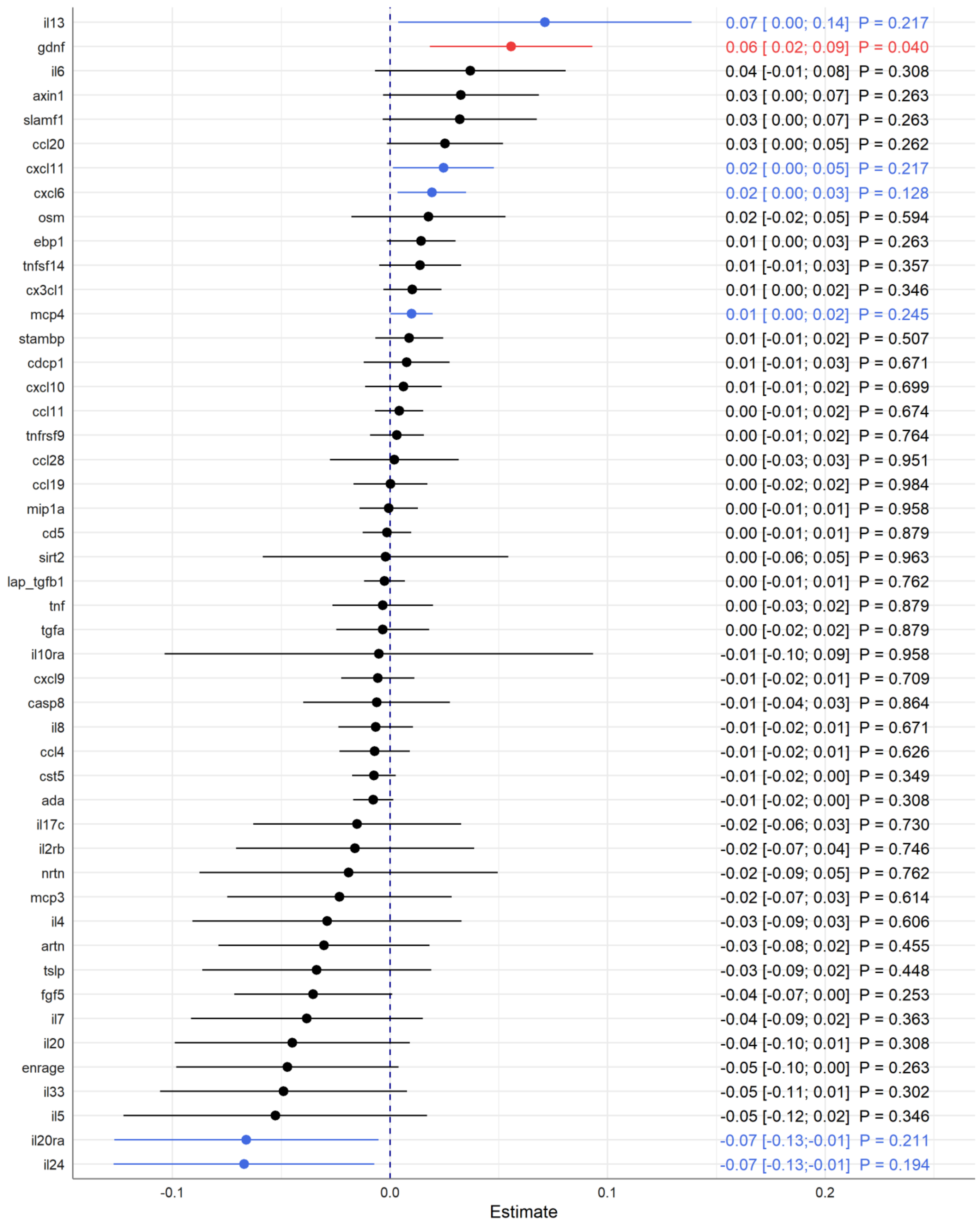

Supplementary Figure 5: Estimates obtained from multivariable log-linear regression models in the KORA-Age1 study.

β coefficients and 95% confidence intervals represent the associations between statin intake and inflammation parameters. P-values are FDR-adjusted. Red and blue colors represent notable associations before and after adjustment for multiple testing.
