# Supplementary material for: Pleiotropic effects between statin intake and inflammation parameters in two distinct population-based studies
Source: Commun Med (Lond). 2025 Sep 11;5:387. doi: 10.1038/s43856-025-01124-x (PMC12426230; doi:10.1038/s43856-025-01124-x)
Supplement: Supplementary file 3 — Description Supplementary Data [file 43856_2025_1124_MOESM3_ESM.pdf]

Description of Additional Supplementary Data Files

Fina name	Description
Supplementary Data 1	Specification of model-specific restricted cubic splines in the KORA-Fit study
Supplementary Data 2	Specification of model-specific restricted cubic splines in the KORA-Age1 study
Supplementary Data 3	Distributions of the inflammation parameters stratified by statin intake in the KORA-Fit study
Supplementary Data 4	Distributions of the inflammation parameters stratified by statin intake in the KORA-Age1 study
Supplementary Data 5	Source data for Figures 1 – 3
