# Supplementary material for: Pleiotropic effects between statin intake and inflammation parameters in two distinct population-based studies
Source: Commun Med (Lond). 2025 Sep 11;5:387. doi: 10.1038/s43856-025-01124-x (PMC12426230; doi:10.1038/s43856-025-01124-x)
Supplement: Supplementary file 9 — Reporting summary [file 43856_2025_1124_MOESM9_ESM.pdf]

nature portfolio

Corresponding author(s): Dennis Freuer

Last updated by author(s): Jun 10, 2025

Reporting Summary

Nature Portfolio wishes to improve the reproducibility of the work that we publish. This form provides structure for consistency and transparency in reporting. For further information on Nature Portfolio policies, see our Editorial Policies and the Editorial Policy Checklist.

Statistics

For all statistical analyses, confirm that the following items are present in the figure legend, table legend, main text, or Methods section.

n/a	Confirmed	
☐	☒	The exact sample size (n) for each experimental group/condition, given as a discrete number and unit of measurement
☐	☒	A statement on whether measurements were taken from distinct samples or whether the same sample was measured repeatedly
☐	☒	The statistical test(s) used AND whether they are one- or two-sided<br>*Only common tests should be described solely by name; describe more complex techniques in the Methods section.*
☐	☒	A description of all covariates tested
☐	☒	A description of any assumptions or corrections, such as tests of normality and adjustment for multiple comparisons
☐	☒	A full description of the statistical parameters including central tendency (e.g. means) or other basic estimates (e.g. regression coefficient) AND variation (e.g. standard deviation) or associated estimates of uncertainty (e.g. confidence intervals)
☐	☒	For null hypothesis testing, the test statistic (e.g. F, t, r) with confidence intervals, effect sizes, degrees of freedom and P value noted<br>*Give P values as exact values whenever suitable.*
☒	☐	For Bayesian analysis, information on the choice of priors and Markov chain Monte Carlo settings
☒	☐	For hierarchical and complex designs, identification of the appropriate level for tests and full reporting of outcomes
☐	☒	Estimates of effect sizes (e.g. Cohen's d, Pearson's r), indicating how they were calculated

Our web collection on statistics for biologists contains articles on many of the points above.

Software and code

Policy information about availability of computer code

Data collection	Data collection in KORA was performed with Microsoft Access (Version 2007 and 2016).
Data analysis	All analyses were conducted using the open-source statistical Software R (version 4.4.0).

For manuscripts utilizing custom algorithms or software that are central to the research but not yet described in published literature, software must be made available to editors and reviewers. We strongly encourage code deposition in a community repository (e.g. GitHub). See the Nature Portfolio guidelines for submitting code & software for further information.

Data

Policy information about availability of data

All manuscripts must include a data availability statement. This statement should provide the following information, where applicable:

- Accession codes, unique identifiers, or web links for publicly available datasets
- A description of any restrictions on data availability
- For clinical datasets or third party data, please ensure that the statement adheres to our policy

KORA data are available on reasonable request according to the terms and conditions of Helmholtz Munich (https://helmholtz-muenchen.managed-otrs.com/external) and subject to the to approval by the KORA board.

Human research participants

Policy information about studies involving human research participants and Sex and Gender in Research.

Reporting on sex and gender	Sex as a biological attribute was determined based on self-reports and used as a confounding factor in the regression models. No sex-specific differences in the outcomes were to be assumed, so that no sex-specific analyses were performed.
Population characteristics	In both studies, the participants who took statins had lower cholesterol levels, but higher levels of HbA1c and were more frequently affected by diabetes, hypertension, myocardial infarction, and stroke [Tables 1 and 2]. Furthermore, statin users in the KORA-Fit cohort tended to be male, were older, and had a higher BMI, whereas they were better educated in the KORA-Age1 cohort.
Recruitment	The KORA (Cooperative Health Research in the Augsburg Region, Germany) study has replaced and further developed the MONICA (Monitoring of trends and determinants in cardiovascular disease) study since 1996. The KORA cohort study consists of four cross-sectional baseline surveys (S1 1984/85, S2 1989/90, S3 1994/95 and S4 1999/2001, S1-S3 under the label MONICA). The population based representative sample was randomly drawn by a residents' registration office. All study participants have been followed up since the baseline surveys. In 2018/2019, the follow-up study “KORA-Fit” was conducted, in which all living participants of the KORA cohort who were born between 1945 and 1964 and agreed to be contacted again were included (n = 3059 or 64.4 % of all eligible participants).
Ethics oversight	The ethics committee of the Bavarian Medical Association approved both studies (KORA-Fit EC no. 17040; KORA-Age EC no. 08064).

Note that full information on the approval of the study protocol must also be provided in the manuscript.

Field-specific reporting

Please select the one below that is the best fit for your research. If you are not sure, read the appropriate sections before making your selection.

☒ Life sciences ☐ Behavioural & social sciences ☐ Ecological, evolutionary & environmental sciences

For a reference copy of the document with all sections, see nature.com/documents/nr-reporting-summary-flat.pdf

Life sciences study design

All studies must disclose on these points even when the disclosure is negative.

Sample size	In the present study, a subgroup of all KORA-Fit study participants was analyzed, namely the participants who took part in both the S4 baseline survey and the KORA-Fit study (n = 856). A total of 803 participants with available data on Olink inflammation parameters could be included in the analysis.
Data exclusions	Participants without measured inflammation parameters were excluded from the analyses. Additionally, proteins with more than 25% of values below the limit of detection and proteins with only missing values were excluded from the analysis. Since the missing values, the proportion of which was low, were assumed to be completely at random (MCAR), a complete case analysis was performed.
Replication	In order to strengthen the evidence through replication, two population-based cross-sectional studies KORA-Fit and KORA-Age1 were considered. Furthermore, in sensitivity analyses, the results were replicated by performing quantile regression models for notable associations (where $\tau = 0.5$). All replication analyses led to consistent results in terms of the direction of point estimates.
Randomization	Confounding factors were identified using a directed acyclic graph (DAG) and selected based on the disjunctive cause criterion. The potential effect of unmeasured confounding was assessed and quantified using the E-value, which is defined as the minimum strength of association from an unmeasured cofounder on the risk ratio scale needed to fully explain away a specific exposure-outcome association.
Blinding	Blinding was not relevant as this was an observational study.

Reporting for specific materials, systems and methods

We require information from authors about some types of materials, experimental systems and methods used in many studies. Here, indicate whether each material, system or method listed is relevant to your study. If you are not sure if a list item applies to your research, read the appropriate section before selecting a response.

Materials & experimental systems

n/a	Involved in the study
☒	☐ Antibodies
☒	☐ Eukaryotic cell lines
☒	☐ Palaeontology and archaeology
☒	☐ Animals and other organisms
☒	☐ Clinical data
☒	☐ Dual use research of concern

Methods

n/a	Involved in the study
☒	☐ ChIP-seq
☒	☐ Flow cytometry
☒	☐ MRI-based neuroimaging
